# Disordered eating attitudes in female students of An-Najah National University: a cross-sectional study

**DOI:** 10.1186/s40337-018-0204-4

**Published:** 2018-08-01

**Authors:** Raghad N. Saleh, Razan A. Salameh, Heba H. Yhya, Waleed M. Sweileh

**Affiliations:** 1 Department of Medicine, College of Medicine and Health Sciences, An-Najah National University, Nablus, 44839 Palestine; 2 Department of Physiology, Pharmacology, and Toxicology, College of Medicine and Health Sciences, An-Najah National University, Nablus, 44839 Palestine

**Keywords:** Female students, Palestine, Eating disorder, SCOFF, EAT-26

## Abstract

**Background:**

Eating disorders (ED) are serious psychiatric disorders characterized by unhealthy eating habits. There is a limited number of studies on eating disorders among female university students in Arab countries. Therefore, the objective of this study was to examine the prevalence of disordered eating attitudes (EA) among female students at An-Najah National University, Palestine.

**Methods:**

A survey study on 2001 female students at An-Najah National University was carried out. The Sick, Control, One Stone, Fat, Food (SCOFF) screening questionnaire and the Eating Attitudes Test (EAT-26) were used.

**Results:**

Of the 2001 participants, 28.6% scored ≥ 20 on the EAT-26 while 38.2% scored ≥ 2 on the SCOFF scale. A significant positive correlation was found between body mass index (BMI) and EAT-26 and SCOFF scores. There was a significant difference in EAT-26 (*p* < .01) and SCOFF scores (*p* = .037) between different academic specializations. Female students in non-scientific fields (arts and humanities) obtained higher scores than female students in scientific/medical fields. Age was significantly and negatively correlated with EAT-26 scores but not with SCOFF scores. Approximately 85% of students with scores in the “high risk” category of the EAT-26 scale endorsed the item “**I am terrified about being overweight**”.

**Conclusion:**

Awareness regarding appropriate nutrition in relation to body weight is needed among female university students. A general university elective course in this regard might be helpful.

## Plain English summary

Eating disorders are common in young females. There is inadequate literature on eating disorders in university students in Arab countries in general, and in Palestinian territories, in particular. A survey of approximately 2000 female students at An-Najah National University using internationally accepted scales showed a relatively high prevalence of disordered eating attitudes. Age, academic specializations, and body weight were important factors associated with disordered eating attitude. The higher the body weight, the higher the risk of having disordered eating attitudes. Younger females had higher disordered eating attitudes than older students. Female university students in non-scientific field had higher disordered eating attitude than those in scientific fields. To combat the influence of media on female university students, educational material regarding health body weight need to be developed and distributed.

## Background

Eating disorders (EDs) are a group of mental illnesses characterized by abnormal eating habits [1]. From a clinical point of view, EDs are an important cause of morbidity and mortality in adolescent girls and young adult women due to the severe changes in their eating behaviors [2, 3]. In the Middle East and in many Arabic countries obesity is prevalent. In fact, the prevalence of overweight and obesity in the Middle East has been estimated to be the second in the world, only after North America [4]. Given that obesity is a major driving force for disordered eating attitudes [5, 6], it is expected that EDs are increasing in Arab populations. A literature search using SciVerse Scopus indicated that there are 19 published articles on eating disorders among women in Arab countries [7, 8]. However, only few were carried out in female university students [9, 10]. Studies from the USA and European countries indicated that female university students had a high prevalence of EDs [11, 12]. University students have several risk factors that increase their risk of eating disorders, such as peer pressure, academic stress, living in dormitories, close relationships, social interaction, and high life expectations [13, 14]. Studies have shown that early detection and treatment of EDs can lead to full recovery [15, 16]. Therefore, screening for EDs in university students using simple and valid scales may allow early detection and appropriate medical and psychological intervention. Examples of useful scales for detection of EDs include the Sick, Control, One stone, Fat, Food (SCOFF) [17–21] and Eating Attitudes Test-26 (EAT-26) [22, 23] which can facilitate screening and early detection of EDs among college students [17, 20].

Based on this background, we hypothesized that the prevalence of EDs in Palestinian female university students is as high as reported in other Arab and non-Arab countries. The results of the current study will add to the literature on ED among Arab female adolescents and young adults, which will allow for comparative analysis with other cultural and ethnic groups, given that cultural differences in EDs do exist [24]. Furthermore, in this study, we will investigate how variables such as body weight, residence (living with family or in student dormitories), and academic specialization might be associated with ED, which may help in tailoring intervention strategies on university campuses.

## Methods

### Participants

Female students in different academic disciplines at An-Najah National University were invited to participate in this study.

### Instruments

The researchers reviewed several articles published among female university students in the Middle East region and developed a questionnaire to be used for the purpose of this survey study. The questionnaire included several demographic variables; such as age, place of living, and academic specialization. The questionnaire also included two commonly used scales; SCOFF and EAT-26 [2, 19, 25]. Each of them is a highly reliable and valid scale and used to assess the risk of eating disorders.

#### SCOFF scale

The SCOFF is a screening questionnaire developed by Morgan et al. [19], which contains five short questions regarding key aspects of eating disorders such as vomiting, concerns about losing control over how much one eats, weight loss, feeling fat and whether food dominates life. The SCOFF scale consists of five yes/no questions. The Arabic version of the SCOFF (A-SCOFF) was obtained from the author who validated this version of the scale [17]. The scoring of SCOFF was as follows: one point for every “yes” answer and 0 for every “no” answer. A total score of ≥ 2 indicates that the participant has a high possibility of having anorexia nervosa or bulimia nervosa [19]. The Arabic version of the SCOFF has a sensitivity of 80.0%, a specificity of 72.7%, and a Cronbach alpha scale reliability coefficient of 0.43 [17]. In this study, Cronbach’s α of the A-SCOFF equaled 0.38.

#### EAT-26 scale

The EAT-26 scale has three subscales with a total of 26 questions: 1) Dieting, 2) Bulimia and Food Preoccupation and 3) Oral Control [25]. Each item (except the 25th item) has six response options ranging in value from 0 to 3 (“always” = 3, “almost always” = 2, “often” = 1, “seldom” = 0, “hardly ever” = 0 and “never” = 0). Item 25 has a reversed score (“always”, “nearly always” and “often” = 0, “seldom” = 1, “almost never” = 2, “never” = 3). The total score of EAT-26 equals the sum of scores for the 26 items. A score of ≥ 20 is defined as being characteristic of a “disordered eating attitude”. In this study, the Cronbach’s α of EAT-26 was 0.87. The Arabic language version of this scale was validated more than two decades ago and found to be valid and reliable [26]. The Arabic version used in the current study was a translation made by the authors to suit the Palestinian Arabic accent and was generated with the help of English and medical professionals.

#### Behavioral scale

The EAT-26 has three criteria for determining if the participant needs to seek further evaluation of the risk of having ED. These criteria include the EAT-26 scores, low body weight compared to age-matched norms, and behavioral questions indicative of possible ED [2]. In the current study, five behavioral questions were added to the EAT-26 to determine the presence of extreme weight-control behaviors as well as providing an estimate of their frequency. These questions assess self-reported binge eating, self-induced vomiting, use of laxatives, over-exercising, and loss of more than 8 kg over the preceding 6 months. According to the guideline, individuals who endorsed any of these items have high potential of having ED and require further assessment by trained mental health specialist [27–30].

### Procedure

This was a cross-sectional study carried out at An-Najah National University, the largest university in Nablus and elsewhere in the Palestinian territories. Approximately 10,000 female students are currently enrolled at An-Najah National University in various academic specializations. This study was carried out during the autumn semester of 2017 and included female students from different specialties in the two main campuses of the university.

The researchers distributed the questionnaires to female students in seven different sites in the university where students from all colleges usually spend their break time. The distribution and collection process were carried out for eight consecutive days with 300 questionnaires being distributed daily. The distribution and collection of the questionnaires were carried out by three co-authors (H.Y, R.N.S and R.A.S).

### Data entry

All data collected were entered into the Statistical Package for Social Science (SPSS) 20 software. The variables included in the study were age, academic specialization (coded as “1” for those in humanities and “2” for those in other fields that included: science, engineering, and medicine), and living situation (coded as “1” for living with family and “2” for living in dormitories). Both height and weight were measured by the researchers using a standard electronic scale and stadiometer obtained from the University. Body Mass Index (BMI) was calculated by dividing body weight in kilograms by the height in meters squared. The following classification was used: BMI ≥ 30.00 indicates obesity, 25.00 ≤ BMI < 30 overweight, 18.5 ≤ BMI < 25 defines the normal range, BMI < 18.5 is considered as underweight [31].

### Ethical approval

The questionnaire was anonymous, to ensure confidentiality. Furthermore, an approval from the institutional review board [32] at An-Najah National University was obtained to carry out the study among female university students. Since no clinical intervention or blood testing was involved in the study, verbal consent, and not a written consent, was obtained from all participants, according to IRB regulations.

## Results

### Characteristics of the participants

In the current study, the researchers distributed approximately 2500 questionnaire. In total, 2001 female students completed and returned the questionnaire, giving a response rate of 97.1%. More than half (1092; 54.6%) of the participants were from faculties of humanities and arts while the remaining (909; 45.4%) were from faculties of medicine or science or engineering. The majority of respondents (1676; 83.8%) were living with their families while the remaining were living in student dormitories. The mean age ± SD of the respondents was 19.5 ± 1.4 years while BMI mean ± SD was 21.7 ± 3.4 kg/m^2^. Based on BMI classification, 1467 (73.3%) of the participants had a BMI in the “normal” range, 281 (14.0%) in the “underweight” range, and 253 (12.6%) in the “overweight/obese” range.

### Eat-26 and SCOFF scores

Table 1 shows Cronbach α value for the scales used and the mean ± SD scores of both SCOFF and EAT-26 subscales. Internal consistency of EAT-26 and its subscales were high, but that of the SCOFF was low. According to the EAT-26 scale, the number of female students considered to be at high risk of disordered eating attitudes was 573 (28.6%), while according to the SCOFF, the number was 767 (38.3%). There was a significant and positive correlation between SCOFF scores and EAT-26 and its subscale scores (Table 2), suggesting a high degree of agreement between the two scales.

**Table 1:** Analysis of SOFF and EAT-26 scores

Variable	Cronbach’s α	Mean ± SD and	Range of scores
SCOFF	0.38	1.25 ± 1.032	0–4
EAT – 26	0.87	15.27 ± 10.38	0–69
D subscale	0.88	8.21 ± 6.977	0–39
O subscale	0.79	4.67 ± 6.977	0–21
B subscale	0.86	2.39 ± 3.272	0–17

**Table 2:** Correlation between SCOFF scores and EAT-26 scores

		EAT-26	D-score	O-scale	B-score
SCOFF scores	Pearson Correlation	0.423^a^	0.404^a^	0.075^a^	0.391^a^
	Sig. (2-tailed)	0.000	0.000	0.001	0.000

^a^denotes significance with *p* value less than 0.01

### Univariate analysis of EAT-26 scores

There was a significant and negative correlation (*r* = − 0.058, *p* = .008) between age and EAT-26 scores suggestive of higher risk of ED among younger female students. There was also a significant and positive correlation between BMI and EAT-26 (Spearman correlation *r* = 0.173, *p* < 0.011) suggestive of higher risk of ED among females with higher BMI values. Females in the academic field of humanities had a mean ± SD on the EAT-26 of 16.4 ± 10.8 while females in science/engineering/medicine had a mean ± SD of 13.9 ± 9.7. The difference was significant (*p* < 0.01). Analysis of EAT-26 items indicated that out of the 573 high-risk individuals, 84.5% (*n* = 484) were “terrified of being overweight”, 48.7% (*n* = 279) reported that “food controls their life”, and 55% (*n* = 315) reported “cutting food into pieces”. In the behavioral scale appended to the EAT-26, more than half (53.5%) did not endorse any of the items, while the remaining 930 (46.5%) endorsed at least one of the additional ED behaviours, which suggests a need for further investigation of potential EDs.

### Univariate analysis of SCOFF scores

There was no significant correlation (*p* = .725) between age and SCOFF scores. However, there was a significant correlation between BMI values and SCOFF scores (*r* = .225, *p* < 0.001) such that females with higher BMI values had higher SCOFF scores, which is consistent with results obtained on the EAT-26. Furthermore, there was a significant difference in SCOFF mean scores between humanities students (1.4 ± 1.0) and those in science/medicine/engineering faculties (1.2 ± 1.0) (*p* = .037).

## Discussion

The aim of this study was to investigate the prevalence of eating disorder attitudes among a large sample of female university students using two common eating disorder scales. The two scales showed similar relationships with most variables except for age, which was significantly correlated with the EAT-26 but not the SCOFF. In the context of this study, the internal consistency of the SCOFF was low, while that of the EAT-26 was very high, suggesting that results obtained from EAT-26 were more reliable than that of the SCOFF, even though the SCOFF is simpler scale than the EAT-26.

When compared with similar studies on female university students carried out elsewhere, the rates of eating disorder attitudes/behaviours in the present study were higher than those obtained in Nigeria [33], Iran [34–36], China [37–39], France [37], USA [3, 40, 41], Spain [42], Lebanon [43], Thailand [44], Greece [45], Brazil [46] but lower than those obtained in Bangladesh [47] and Vietnam [48]. Therefore, our results show higher rates than most of those from other countries. The results reported in the current study were within the range of findings from other Arab countries, including Palestine. The reported percentage of girls and young women at risk of ED in Saudi Arabia was 24.6% [26], 29.4% in Oman [49], 23.4% in the UAE [50], and approximately 38% in Palestine 38.9% [14].

The finding of higher rates in this study could be attributed to the new evolving life of Palestinians who have had several decades of hardship. The situation in the Palestinian territories is very complex with regard to exposure to the westernized secular Israeli culture. While female Palestinian students generally have limited exposure to the Israeli community, female Palestinian students from Jerusalem and Israeli Arab students studying at An-Najah National University are exposed to Israeli culture. Such exposure could be affecting views regarding body image and body weight. Palestinian females who are not directly exposed to the Israeli community still experience exposure to Israeli media through television, social media, and other means of communication. Furthermore, recently there has been increased exposure to Turkish media. Such movies and TV shows may also influence the attitudes of Arab females, given that Turkish society is less conservative compared to that of Arab culture. Palestinian culture has been oppressed for decades, and isolated from most direct means of communication with neighboring Arab countries. However, recent communication through social media may have created a conflict in attitudes toward women, female bodies, and beauty standards. The traditional Palestinian culture gives little attention to body weight and what the female body should look like. However, partly due to the above, there has been an increased emphasis on body weight as an important aesthetic item.

The current study showed that there was a significant correlation between BMI and both EAT-26 score and SCOFF score. This was unsurprising, as similar findings have been reported in other studies [51–53]. The current study also showed that academic specialization in faculties related to humanities was significantly associated with high EAT-26 scores as well as high SCOFF score. This finding, o the authors’ knowledge, has not been reported previously in the literature. One interpretation of this finding is that female humanities students tend to have more free time to watch television advertisements and use social media, as well as for activities focusing on beauty and body image than students in science- and engineering-based fields, who tend to experience higher academic pressure and less free time. Students in medicine, engineering, and science have usually obtained high grades in high school and have high academic and professional ambitions, which tend to limit their emphasis on body weight, and use of social media regarding issues pertaining to beauty and body image.

The results of the current study revealed that the most frequently endorsed item was “**I am terrified about being overweight**” which is in the EAT-26 Dieting subscale. This indicates that female students are highly conscious about their body weight. There may be several reasons for this, such as media which shows the ideal female body as being thin. Furthermore, there is peer, cultural, and family pressure on females to be accepted socially based on modern criteria. On the Bulimia and Food Preoccupation subscale, the current study revealed that the most highly endorsed item was “I find myself preoccupied with food”, whereas for the Oral Control Subscale, this item was “I cut my food into small pieces”. These findings suggest that the majority of female students in the high-risk category endorsed behaviours more closely aligned with bulimia nervosa than anorexia nervosa. In Middle Eastern cultures, families tend to pressure girls and young women to eat, but also expect them to maintain an appropriate body weight and body size for marriage. These societal pressures may contribute to bulimia nervosa-like behaviours in young female students at a critical age.

This study has limitations that need to be mentioned. First, the convenience sampling method is not random, however the sample size of the study was relatively large compared with other related studies carried out in the Middle East region. Second, the scales used in this study cannot provide a diagnosis, and the lack of mental health assessment by experts made our study valuable only as an initial screening method. Hence, the true prevalence of various types of eating disorders in the Palestinian community of female students needs to be further investigated. The current study should be followed up with larger numbers of students; future studies should also take into consideration religion and level of religiosity as an important variable when investigating eating disorders. Furthermore, the extent of exposure of female students in this geographical region to Israeli community values needs to be considered when designing future studies about eating disorders.

## Conclusion

This study showed suggests that (1) the prevalence of disordered eating attitudes is relatively high among female Palestinian university students, (2) disordered eating attitudes were significantly associated with BMI and academic specialization, and (3) even though both the EAT-26 and the SCOFF showed similar results, the EAT-26 appears to be more reliable when studying university students. These findings suggest the need for clinical screening of eating disorders and medical treatment if needed. Future epidemiological studies that take into consideration a greater number of demographic and psychological variables such as stress, spirituality and religiosity are recommended.
